# Effect of Chitosan, *Aloe vera* Gel and Coconut Oil Edible Coatings on Postharvest Quality and Shelf Life of Strawberry (*Fragaria × ananassa Duch*) Under Ambient Storage

**DOI:** 10.1002/fsn3.71949

**Published:** 2026-05-27

**Authors:** Gazi Mohammad Tasnimul Karim, Munmun Nahar, Soheli Sultana, Mrityunjoy Biswas

**Affiliations:** ^1^ Department of Food Engineering Jashore University of Science and Technology Jashore Bangladesh

**Keywords:** *Aloe vera* gel, chitosan, coconut oil, postharvest quality, shelf life, strawberry

## Abstract

Strawberries (Fragaria *× ananassa*) are highly perishable due to their delicate texture, high respiration rate, and susceptibility to microbial spoilage. This study evaluated the effects of edible coatings chitosan (CH), *Aloe vera* gel (AVG), and coconut oil (CO), applied individually and in combinations, on postharvest quality of strawberries stored under ambient conditions (26°C ± 2°C; 65%–70% RH). Among all treatments, CH + AVG was the most effective, reducing weight loss by 37.98%, preserving firmness by 91.58%, and lowering total plate count by 44.98% compared to the control. This combination also better maintained pH, titratable acidity, TSS, color, phenolic content, and antioxidant activity. AVG alone effectively reduced weight loss and firmness loss, while CH better suppressed microbial growth. CO alone showed limited efficacy but improved when combined with CH or AVG. Overall, uncoated strawberries retained quality for 2 days, whereas CH + AVG extended shelf life to 4 days under ambient storage conditions.

## Graphical abstract

Postharvest application of eco‐friendly edible coatings consisting of chitosan solution (CH), *Aloe vera* gel (AVG), and coconut oil (CO) on strawberry fruit under ambient conditions. Six distinct formulations (CH, AVG, CO, CH + AVG, CH + CO, and AVG + CO) along with a distilled water (control) were analyzed. These treatments significantly preserved physicochemical attributes (weight loss, firmness, color, pH, TA, TSS, and ripening index), maintained total phenolic content and antioxidant activity, and suppressed disease incidence and microbial growth. Advanced analyses, including PCA, correlation, heat maps, Random Forest classification, and TOPSIS analysis, confirmed that these coatings effectively optimize postharvest quality and extend fruit shelf life during storage.

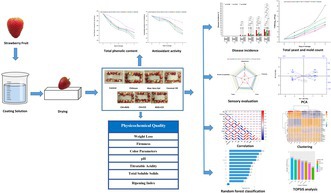

## 1 Introduction

Strawberry is a staple fruit for millions of people worldwide. It is a stoloniferous, perennial plant, scientifically known as *Fragaria × ananassa*, a member of the Rosaceae family. This fruit is an abundant supply of vital nutrients, encompassing vitamins, minerals, flavonoids, anthocyanins, proteins, and phenolic compounds (Kishimoto et al. 2023). Strawberries are particularly perishable climacteric fruits. Their delicate texture, substantial moisture content, and accelerated respiration rates result in a significantly restricted shelf life of merely 1–2 days at ambient temperature and 5–7 days when refrigerated (da Silva Simões et al. 2025; Popescu et al. 2022). Moreover, mechanical damage and vulnerability to opportunistic pathogens, particularly fungi, accelerate postharvest senescence, leading to rapid decay and significant deterioration in essential characteristics such as color and texture (Azodanlou et al. 2003; Ladika et al. 2024).

To alleviate these losses, edible coatings have emerged as a safe and sustainable solution that creates a thin, protective layer on fruit, thereby reducing water loss, respiration, and mold development, which helps maintain strawberries' firmness, brightness, flavor, and nutritional value for extended periods during storage and transportation (Pinzón et al. 2019; De Albuquerque Sousa et al. 2024; Moghadas et al. 2024; Popescu et al. 2022; De Bruno et al. 2023; Alharaty and Ramaswamy 2020; Priyadarshi et al. 2024). Chitosan is extensively employed as a biopolymer in numerous industrial applications and is renowned for its biodegradability as well as its potent antifungal and antibacterial properties (Oberlintner et al. 2021; Sen et al. 2022; Garcia et al. 2020; Hasheminejad et al. 2019; Bi et al. 2021; Song et al. 2022). *Aloe vera* gel creates a thin, imperceptible barrier on the fruit's surface that diminishes water loss, respiration, and softening, thereby preserving firmness, color, vitamin C, antioxidant activity, and inhibiting microbial growth and fungal decay (e.g., *Botrytis cinerea*) during storage (Rehman et al. 2022; Nasrin et al. 2017; Shahdadi et al. 2025; Rahimi 2024; Sogvar et al. 2016), while also reducing microbial growth. Coconut oil has also been investigated; it creates a protective barrier between lenticels and stomata, resulting in decreased respiration and transpiration rates, which ultimately restricts microbial development and activity (Nasrin et al. 2020).

Recent literature has highlighted the efficacy of various targeted postharvest treatments for maintaining strawberry quality. The postharvest treatment with calcium chloride (CaCl_2_) effectively reduces decay rates and preserves phenolic compounds by structurally reinforcing the cell wall (Eroğlu et al. 2024). Exogenous polyamine treatments, including spermidine, have demonstrated significant efficacy in delaying metabolic senescence and preserving physicochemical quality during cold storage (Orman et al. 2024). Nonetheless, whereas these single‐target chemical and biochemical elicitors demonstrate considerable effectiveness, the novelty of the current study resides in the investigation of a wholly natural composite. Unlike chemical dips that primarily target internal metabolic pathways or structural cross‐linking, our study uniquely evaluates the synergistic physical and antimicrobial mechanisms of a composite edible coating, particularly its capacity to concurrently establish a respiration‐limiting gas barrier and physically disrupt microbial membranes without employing synthetic or targeted chemical agents.

This study aimed to assess the individual and synergistic effects of edible coatings on prolonging the shelf life, delaying visual disease onset, and preserving the physicochemical properties, bioactive compounds, antioxidant activity, and microbial stability of stored strawberries, given their highly perishable nature and the necessity for comprehensive preservation methods.

## 2 Materials and Methods

### 2.1 Experimental Location and Biological Material (Strawberry)

All experiments were conducted in late February at the Department of Food Engineering (FE) laboratory facilities at Jashore University of Science and Technology (JUST), Bangladesh, under normal laboratory conditions (average temperature 24°C–28°C). Strawberry seedlings (Fragaria × ananassa Duch.) cv. RU‐1 (Festival) were obtained from Akafuji Nursery (Rajshahi, Bangladesh) and subsequently cultivated and harvested at a local farm in Jashore, Bangladesh, and carefully transported to the laboratory in cushioned cork‐sheet boxes to minimize mechanical damage prior to analysis.

### 2.2 Experimental Design

The experiment was conducted using a completely randomized design (CRD) with seven coating treatments: control, chitosan (CH), *Aloe vera* gel (AVG), coconut oil (CO), CH + AVG, CH + CO, and AVG + CO under ambient storage conditions (26°C ± 2°C). Strawberries were dipped in the respective coating solutions for 20–30 s and air‐dried at room temperature for 3 h before storage. A total of 784 physiologically mature strawberry fruits of uniform size and maturity were utilized for this study. For each treatment, the fruits were divided into seven independent batches (biological replicates), with each batch containing 16 fruits. Three batches were dedicated to non‐destructive evaluations (such as disease incidence). The remaining four batches were allocated for destructive analyses, from which four fruits per treatment were randomly selected on each evaluation day. The fruits were stored under ambient conditions and evaluated at 1‐day intervals. A 25% disease incidence threshold was established as the limit for commercial marketability; data collection for each treatment group was terminated once this threshold was exceeded. Analytical measurements were performed with technical replicates, and results were expressed as mean ± standard deviation.

### 2.3 Coating Preparation

The coating solutions were prepared following the method described by Begum et al. (2023), with slight modifications.

#### 2.3.1 Preparation of CH Coating

A 0.5% solution of 100 mL of acetic acid was used to dissolve 1 g of CH (low molecular weight, 90% deacetylation degree, 10–150 mPa·s, extra pure grade, Sisco Research Laboratories Pvt. Ltd., India) in order to create a 1% CH solution. Using a digital hot plate and a magnetic stirrer, the liquid was homogenized, the temperature was maintained at 70°C for 40 min, and NaOH was added to bring the pH to 5.6. Four layers of cotton cloth were then used to filter the fluid. Next, 2% glycerol was added as a plasticizer to the mixture.

#### 2.3.2 Preparation of AVG Coating

*Aloe vera* leaves were first cleaned with distilled water (DW) to remove surface debris. The leaves were then sliced to extract aloin, a yellow liquid. A lab‐scale roll processor was used to separate the outer green layer of the *Aloe vera* leaf, after carefully separating the outer green layer from the parenchyma (inner part containing the gel) with a lab‐scale roll processor. This extraction process yielded approximately 38 g of pure *Aloe vera* gel per 100 g of fresh leaves. Then the gel was blended, filtered through a muslin cloth, and mixed with 1% and 0.5% w/v citric and ascorbic acid as preservatives. To complete the process, the mixture was pasteurized in a beaker, heated to 70°C for 40 min, and, at the end of the heating period, 2% glycerol was added as a plasticizer.

#### 2.3.3 Preparation of CO Coating

CO (100% pure natural; cold‐pressed; Laxzin, Bangladesh) was prepared in a 1:1 (v/v) ratio with DW, incorporating 2% Tween 80 as a surfactant and 2% glycerol as a plasticizer. The CO solution is then continuously stirred with a magnetic stirrer for 30 min.

#### 2.3.4 Preparation of CH + AVG Coating

The mixed solutions of CH and AVG were made by adding 1:1 v/v of the CH and AVG solutions. A magnetic stirrer was used to mix the CH solution with the AVG solution. A magnetic stirrer was used to mix the CH and AVG solutions for 1 h at ambient temperature.

#### 2.3.5 Preparation of CH + CO Coating

The CH and CO coating solutions were mixed in a 1:1 volume ratio in a beaker. Subsequently, 2% Tween 80 was added as an emulsifying agent to facilitate mixing of the CH solution and oil. The mixture was then heated on a hot plate and continuously agitated with a magnetic stirrer for 1 h. Afterward, 2% glycerol was included as a plasticizer.

#### 2.3.6 Preparation of AVG + CO Coating

The complete mixture of the CO coating solution with the AVG coating solution was mixed in a 1:1 volume ratio in a beaker. Then, 2% Tween 80 was added as an emulsifying agent to properly mix the AVG solution and oil. The mixture was subsequently heated on a hot plate and stirred continuously with a magnetic stirrer for 1 h; thereafter, 2% glycerol was added.

### 2.4 Experimental Treatments

The coating formulations are summarized in Table 1.

**TABLE 1:** Strawberries were treated with six different coatings, along with a control.

Treatment NO.	Coating name	Composition for application	Key preparation steps (for single component)	Key additives/mixing (for final solution)
i.	Control	DW	N/A	N/A
ii.	CH	1% CH solution (w/v)	Dissolved in acetic acid (0.5%), Homogenized (70°C, 40 min), pH 5.6.	Glycerol (2%)
iii.	AVG	AVG (1:1, v/v)	Cleaned, aloin removed, gel separated, Blended, filtered, pasteurized (70°C, 40 min)	1% and 0.5% w/v citric and ascorbic acid Glycerol (2%)
iv.	CO	CO (1:1, v/v)	CO in DW (1:1 v/v), Stirred (30 min)	Tween 80 (2%), Glycerol (2%)
v.	CH + AVG	1% CH + AVG (1:1, v/v)	N/A (Solutions pre‐prepared)	CH Solution + AVG Solution (1:1 v/v), Magnetic Stirring for 1 h at ambient temperature
vi.	CH + CO	CH + CO (1:1, v/v)	N/A (Solutions pre‐prepared)	CH solution + CO solution (1:1 v/v), 2% Tween 80, magnetic stirring for 1 h, 2% glycerol added afterwards
vii.	AVG + CO	AVG + CO (1:1, v/v)	N/A (Solutions pre‐prepared)	AVG solution + CO solution (1:1 v/v), 2% Tween 80, magnetic stirring for 1 h, 2% glycerol added afterwards

### 2.5 Parameters Studied

#### 2.5.1 Weight Loss

A top pan electric balance was used to weigh each strawberry at 1‐day intervals to calculate weight loss, a method described by Costa et al. (2011). Each treatment involved sixteen (16) fruits per batch, which were used consistently throughout the experiment. The weight decrease % was computed at daily intervals during storage using the following Formula (1).

(1) $$\mathrm{WL}\left(\%\right)=\left(\frac{M_{0}-M_{t}}{M_{0}}\right)\times 100$$

where *M*_0_: initial weight (g), *M*_t_: sample's weight.

#### 2.5.2 Firmness

The majority of techniques for determining the hardness of fruits and berries include puncturing or penetrating the fruit with a plunger or needle. The diameter ranges between roughly 3 and 20 mm. The plungers are primarily cylindrical. By using a penetrometer (a 5‐mm diameter puncture probe, round‐tipped), the strawberry was subjected to a firmness test in which at least three (3) measurements at different locations on each fruit, with three fruits, were tested (Døving et al. 2005).

#### 2.5.3 pH

The pH of the samples was measured following the method described by Alam et al. (2023), with slight modifications, using a Hanna Instruments HI‐2211 pH meter. A 5 g sample was homogenized with 45 mL of DW for 30–60 s to obtain a uniform slurry. The pH meter was calibrated prior to measurement, and approximately 25 mL of the homogenized sample was transferred to a beaker. The electrode was then immersed in the sample, and the pH value was recorded once a stable reading was obtained.

#### 2.5.4 Titratable Acidity (TA)

TA of the strawberry samples was determined according to the Association of Official Analytical Chemists (AOAC) (2016) method. A 10 mL aliquot of the homogenized sample was transferred into a conical flask and titrated against standardized 0.1 N NaOH using 2–3 drops of phenolphthalein as an indicator until a persistent pink endpoint was observed. The gram equivalent of citric acid was taken as 64.02, and TA was calculated using the following Formula 2.

(2) $$\begin{array}{c} \text{Titratable acidity}\;\left(\%\right)\\ =\frac{\text{Titre}\times 0.1N\times \text{Volume made}\times \text{Equivalent}\;\mathrm{wt}.\;\text{of acid}\times 100}{\text{The volume of sample}\;\left(\;\text{taken for estimation}\right)\times \mathrm{Wt}.\text{of sample}\times 1000} \end{array}$$

#### 2.5.5 Total Soluble Solids (TSS)

A digital refractometer was used to determine the TSS (°Brix) of the fruit flesh juice (Selcuk and Erkan 2014). The analysis was conducted using the residual juice obtained from the pH measurement. Prior to analysis, the refractometer was calibrated with DW to obtain a zero reading. Subsequently, one to two drops of filtered juice were placed on the prism surface to measure TSS. The initial TSS values of the fruit tissues were calculated by correcting the readings based on the applied dilution factor.

#### 2.5.6 Ripening Index (TSS/TA)

In order to determine the ripening index of treated strawberry, the TSS were divided by the TA (Tinebra et al. 2021).

#### 2.5.7 Color Parameters

To analyze the color parameters of the strawberry in triplicate, a Chroma Meter was used to measure them accurately. Before measurement, the Chroma Meter was accurately calibrated using a standard reference tile. After the samples were collected for measurement, they were positioned on the Glass Light‐Projection tube. The L*a*b* color space operates on a color opposition model, which indicates that two colors, such as red and green, or yellow and blue, cannot appear simultaneously. This is demonstrated below and calculated using the Formula 3.

(3) $$\;\begin{array}{c} ∆{E^{*}}_{\mathrm{ab}}=\sqrt{{\left({L^{*}}_{2}-{L^{*}}_{1}\right)}^{2}+{\left({a^{*}}_{2}-{a^{*}}_{1}\right)}^{2}+{\left({b^{*}}_{2}-{b^{*}}_{1}\right)}^{2}}\\ =\sqrt{{\left(\Delta L*\right)}^{2}+{\left(\Delta a*\right)}^{2}+{\left(\Delta b*\right)}^{2}} \end{array}$$

where Lightness‐darkness is indicated by L*, the red‐green axis by a* and the yellow‐blue axis by b*.

#### 2.5.8 Disease Incidence (Percentage of Infected Fruits)

Disease incidence was assessed based on the time required for visible natural infection to cover 10% of the fruit surface area (Terry and Joyce 2004). Fruits from each treatment were inspected daily for disease symptoms, and incidence data were recorded, with the first assessment conducted on the first day of storage. Disease identification was carried out by observing the characteristic symptoms of gray mold, Rhizopus soft rot, and leather rot, caused by *Botrytis cinerea, Rhizopus stolonifer*, and *Phytophthora cactorum*, respectively (Maas 1998). Disease incidence was determined by counting the number of strawberries showing fungal infection, following Equation 4.

(4) $$\%\text{Disease incidence}=\frac{\text{Number of infected fruits}}{\text{Total number of fruits assessed}}\times 100$$

#### 2.5.9 Determination of Total Phenolic Content (TPC)

The TPC of the strawberry sample was assessed utilizing the Folin–Ciocalteu colorimetric method as outlined by Wootton et al. (2011). Before the analysis, the Folin–Ciocalteu reagent was diluted with DW at a 1:10 (v/v) ratio, and a sodium carbonate solution was prepared by dissolving 7.5 g of Na_2_CO_3_ in 100 mL of DW. For the assay, 5 mL of the diluted Folin–Ciocalteu reagent and 4 mL of the sodium carbonate solution were combined with 1 mL of the ethanolic extract. The resulting mixture was vortexed for 15 s and incubated at 40°C for 30 min to facilitate color development. Absorbance was recorded at 765 nm using a double‐beam UV–Vis spectrophotometer, with DW serving as the blank. The absorbance values were compared with those of gallic acid standard solutions, and the TPC was reported as mg gallic acid equivalents (GAE) using the calibration equation *y* = 0.0074*x* + 0.4075 (*R* ^2^ = 0.9982). The results were presented in mg GAE per 100 g of fresh weight.

#### 2.5.10 Total Antioxidant Capacity via DPPH Radical Scavenging Activity

The DPPH radical‐scavenging activity of the samples was assessed and expressed as percentage inhibition, as described by Ferdaus et al. (2020). An equal volume of 0.1 mM DPPH solution was combined with 2 mL of the sample solution, which consisted of 0.4 mL of ethanolic extract and 1.6 mL of ethanol. The resulting mixture was vortexed for 15 s and subsequently incubated at room temperature in the dark for 30 min to facilitate the reaction and the formation of the characteristic purple color. Absorbance was recorded at 517 nm using a double‐beam UV–Vis spectrophotometer, with ethanol and 0.1 mM DPPH solution as the blank and reference, respectively. % of inhibition was calculated using the following formula (5).

(5) $$\;\text{Inhibition}\;\left(\%\right)=\left(\frac{A_{c}-A_{S}}{A_{c}}\right)\times 100$$

where $A_{c}$ = Control absorbance and $A_{S}$ = Sample absorbance.

#### 2.5.11 Total Yeast and Mold Count

5 g of strawberries were diced into small pieces and suspended in 45 mL of peptone water to evaluate the quantity of yeast and mold. A blender was used to mix the suspension for 5 min. The strawberry homogenates were subjected to serial dilutions (10^−1^ to 10^−6^) and subsequently plated on Potato Dextrose Agar, a selective growth medium. The plates were incubated for 5 days at 25°C without inverting. The yeast and mold counts were reported as log CFU g^−1^, as described by Pinzón et al. (2019), with minor adjustments.

### 2.6 Sensory Analysis

Customer approval of strawberries was evaluated using a nine‐point hedonic scale, where we had verified that the experiment complied with the specified ethical procedures before the sensory evaluation. All subjects gave their consent before the experiment began. Then, 30 randomly selected volunteers aged 20–35 completed this step by giving each participant a blind sensory assessment sheet and asking them to provide a sensory score. The qualities of appearance, fineness, color, taste, and general acceptability were then assessed using a 9‐point hedonic scale.

## 3 Statistical Analysis

All parameters were measured, and the experimental data were subjected to thorough statistical analysis. Data were analyzed using a two‐way analysis of variance (ANOVA) to evaluate the effects of coating treatment and storage time (two factors) and their interaction. When significant differences were observed, mean comparisons were performed using Tukey's honestly significant difference (HSD) test at a significance level of *p* < 0.05. Statistical analyses, including ANOVA, interaction plots, and graphical representations, were performed using OriginPro 2021. Multivariate analyses, including principal component analysis and Pearson's correlation analysis, were also conducted using the same software. Additionally, R statistical software (version 4.4.2) was used for advanced analyses, including hierarchical clustering, random forest classification, and the Technique for Order Preference by Similarity to Ideal Solution (TOPSIS) for treatment ranking.

## 4 Results and Discussion

### 4.1 Parameters Studied

#### 4.1.1 Weight Loss

**FIGURE 1:**
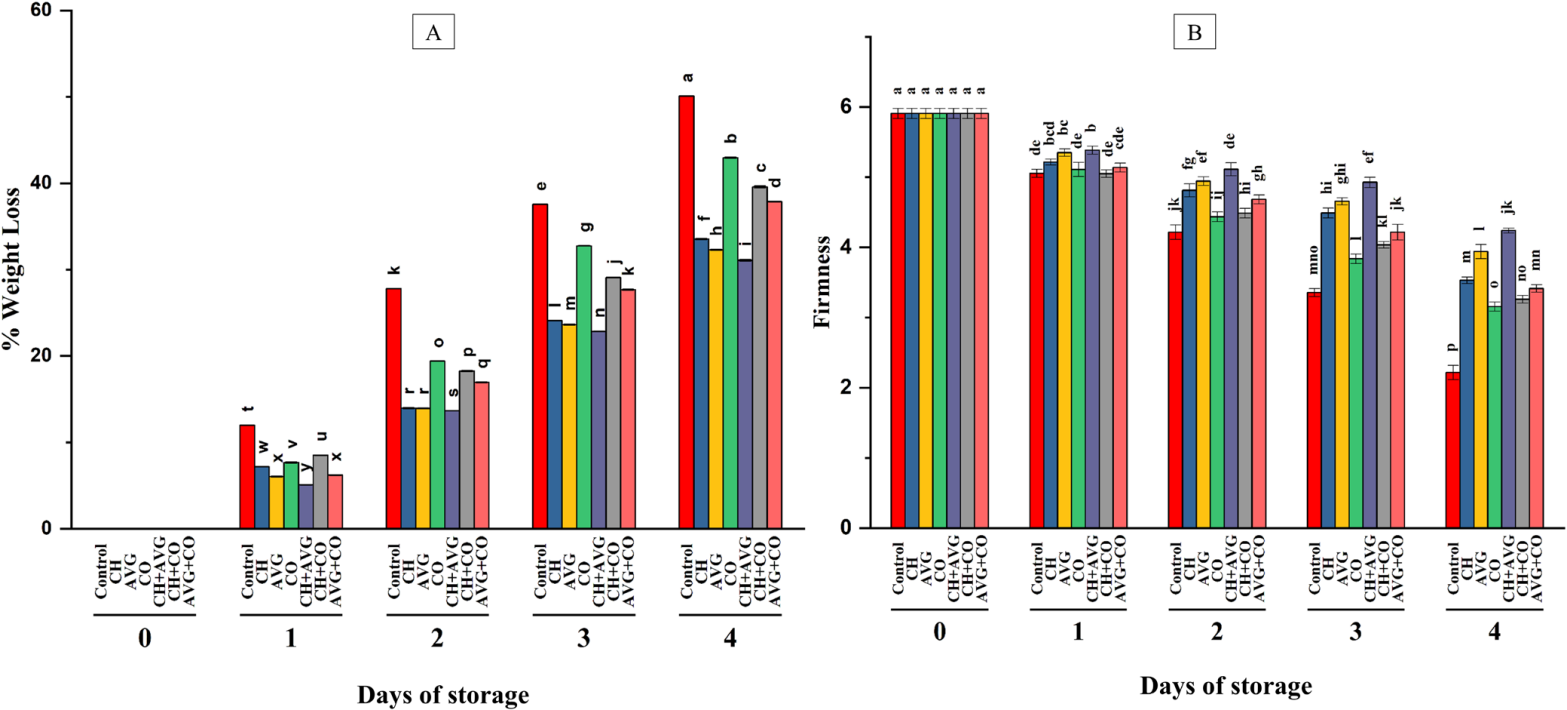
Effects of coatings on weight loss and firmness of strawberry fruit during storage (26°C ± 02°C; 65%–70% RH). Results expressed as mean ± SD (*n* = 3); means sharing identical letters are not significantly different based on two‐way analysis of variance (ANOVA) followed by Tukey's HSD test (*p* < 0.05). AVG + CO, *Aloe vera* gel + coconut oil, AVG, *Aloe vera* gel, CH + VG, chitosan + *Aloe vera* gel, CH + CO, chitosan + coconut oil, CH, chitosan solution, CO, coconut oil, Control, distilled water.

The percentage of weight loss in strawberries during storage varies significantly, as illustrated in Figure 1. Strawberries gradually lose weight while in storage, and the application of coating treatments notably reduces weight loss compared to uncoated strawberries. This is because edible coatings slow down dehydration and delay fruit senescence by creating a physical barrier that minimizes water evaporation (Almenar et al. 2007). The weight loss observed in strawberries treated with CH + AVG (31.06% ± 0.1%), AVG (32.29% ± 0.07%), and CH (33.53% ± 0.06%) was significantly lower. In contrast, greater weight loss was recorded in AVG + CO (37.86% ± 0.06%), CH + CO (39.57% ± 0.12%), and CO (42.95% ± 0.10%) by the end of storage. This is attributed to CO, which may have reduced the ability to form a barrier on strawberries, due to its poor adhesion to the hydrophilic fruit surface and partial melting at ambient temperature, which creates microscopic cracks that allow water vapor to escape easily. The highest weight loss was recorded for uncoated fruits at 50.08% ± 0.04%. The CH + AVG coating consistently resulted in the least weight loss, while uncoated strawberries experienced the most. Furthermore, the weight loss of strawberries treated with CH + AVG was 19.02% lower than that of uncoated strawberries at the conclusion of storage. The diminished weight loss in strawberries coated with edible films is due to the formation of a physical barrier on the fruit's surface, which decreases water evaporation, mitigates dehydration, and therefore postpones fruit senescence (Almenar et al. 2007). Hernandez‐Munoz et al. (2006) and Almenar et al. (2007) reported that edible coatings reduce weight loss in fresh strawberries by creating a physical barrier that restricts moisture evaporation and delays senescence, which are otherwise influenced by respiration and storage conditions. Previous studies indicate that AVG enhances tomato water retention when used in combination with other coatings (Chauhan et al. 2015). According to Duan et al. (2019), the CH coating reduced weight loss in mango fruit without affecting its flavor, aroma, or digestibility. Our results are consistent with previous findings, as CH + AVG has been shown to significantly reduce fruit weight loss during storage at room temperature (Shah and Hashmi 2020; Seyed et al. 2021).

#### 4.1.2 Firmness

One significant quality metric that indicates the strength of the fruit's cell wall and intercellular adhesion is firmness (Huang et al. 2023). The study's findings (Figure 1) reveal that coatings and storage duration significantly influence fruit firmness. The experimental results indicate that edible coatings effectively preserve strawberry firmness when stored under ambient conditions. With the initial hardness of the strawberries measured at 5.91 N, those coated with CH + AVG (4.24 ± 0.03 N) and AVG (3.94 ± 0.09 N) exhibited the highest firmness at the conclusion of the storage period, followed by CH (3.53 ± 0.04 N), AVG + CO (3.41 ± 0.05 N), CH + CO (3.26 ± 0.05 N), CO (3.15 ± 0.06 N) and uncoated strawberries diminished by 62.60% (2.21 N). The gradual decline in strawberry firmness is mostly due to moisture loss and the enzymatic breakdown of cell wall constituents during storage (Hassani et al. 2025). Furthermore, CO was ineffective at maintaining firmness compared to other individual coatings, as it failed to establish a continuous gas barrier, allowing rapid cellular respiration and the activity of pectin‐degrading enzymes to further soften the fruit tissue. Edible coatings, such as AVG coatings, diminish water loss and postpone softening in high‐moisture fruits like strawberries by creating a semi‐permeable layer that blocks surface pores, reduces transpiration and gas exchange, and decelerates ripening and metabolic processes, thus maintaining cellular turgidity (Haider et al. 2022; Sogvar et al. 2016). According to Hernandez‐Munoz et al. (2006), strawberry fruits with organic coatings, such as CH, remain firmer compared to those without any coating. This may be due to its modification of the fruit's internal environment and its decrease in respiration rate, as it functions as a selective gas barrier to O_2_ and CO_2_. Consistent with Begum et al. (2023) for mangoes, our results further indicate that CH coatings, and their combinations with AVG, successfully delayed strawberry softening.

#### 4.1.3 Color Parameters of Skin

**FIGURE 2:**
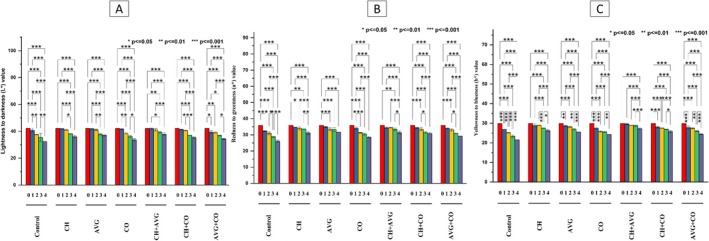
Effects of coatings on color parameters (L*a*b*) of strawberry fruit during storage periods; L* = (A), a* = (B), b* = (C) during storage; L* = lightness (black to white); a* = redness (+) to greenness (−); b* = yellowness (+) to blueness (−); * (*p* ≤ 0.05) → statistically significant, ** (*p* ≤ 0.01) → highly significant, *** (p ≤ 0.001) → very highly significant. AVG + CO, *Aloe vera* gel + coconut oil, AVG, *Aloe vera* gel, CH + VG, chitosan + *Aloe vera* gel, CH + CO, chitosan + coconut oil, CH, chitosan solution, CO, coconut oil, Control, distilled water.

The color of fruits and vegetables significantly affects customer preferences and offers important insights into various other characteristics of the final food product (Yildiz et al. 2019). The lightness (L*) of strawberries consistently decreased during storage, as illustrated in Figure 2A. Compared to the coated strawberries, the uncoated control group exhibited a markedly lower L* value. Strawberries treated with CH + AVG recorded the highest lightness value (L* = 37.82 ± 1.07), which diminished by 10.25% by the end of the storage period, with the treatments ranked as follows: CH + AVG > AVG > CH>CH + CO>AVG + CO, while fruit dipped in CO resulted in significantly reduced lightness. Shafique et al. (2023) noted a similar reduction in the L* of strawberries during storage. The chroma a* value is directly associated with the anthocyanin concentration in strawberries (Virgen‐Ortiz et al. 2020). All treatments exhibited a gradual decrease in a* values (Figure 2B). The control group experienced the most rapid and significant decline in redness, with a* value of 26.03 ± 0.60 on Day 4. In contrast, strawberries treated with CH + AVG showed the best preservation of red color among all treatments, maintaining the highest a* value of 31.47 ± 0.55 at the end of storage, indicating that this combination was the most effective at preserving the red hue. Although not as effective as the CH + AVG combination, strawberries treated with CH and AVG separately also showed a commendable level of color retention. Hernández‐Muñoz et al. (2008) indicated that a shift towards less vibrant colors is reflected in a decrease in chroma values. All treatments showed a significant difference in b* value (Figure 2C), with uncoated strawberries exhibiting the lowest b* value (21.51 ± 0.09). Strawberries coated with CH + AVG achieved the highest b* values (b* = 27.21 ± 0.50). Additionally, coatings based on CO (CO, CH + CO, and AVG + CO) demonstrated reduced effectiveness in preserving the L*a*b* values. Alterations in strawberry color during storage, especially at elevated temperatures, promote senescence, induce membrane damage, and expose substrates to polyphenol oxidase (PPO), resulting in a deeper, brownish hue. Decreased red fruit with diminished anthocyanin levels. Furthermore, PPO and peroxidase (POD) facilitate the synthesis of brown and yellow polymeric pigments through oxygen interaction, thereby reducing color purity and gloss. Elevated temperatures (20°C–35°C) exacerbate color degradation, weight loss, reactive oxygen species accumulation, and enzyme activity (Peng et al. 2017; Lv et al. 2022; Muley et al. 2021; Zhang et al. 2019; Nunes et al. 2005).

#### 4.1.4 Disease Incidence

**FIGURE 3:**
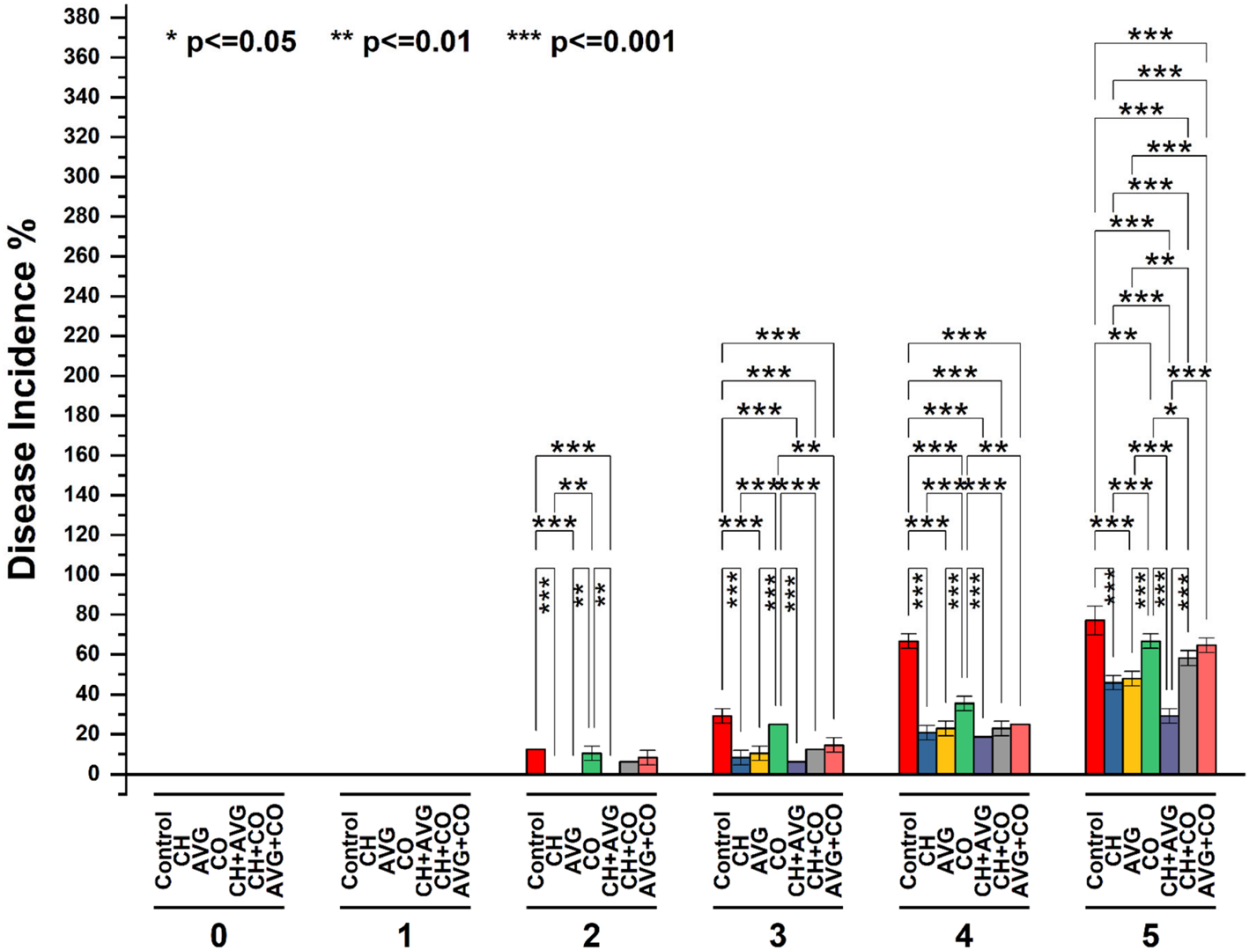
Effects of coatings on disease incidence of strawberry fruit during storage periods; * (*p* < = 0.05) → statistically significant, ** (*p* < = 0.01) → highly significant, *** (*p* < = 0.001) → very highly significant. AVG + CO, *Aloe vera* gel + coconut oil, AVG, *Aloe vera* gel, CH + VG, chitosan + *Aloe vera* gel, CH + CO, chitosan + coconut oil, CH, chitosan solution, CO, coconut oil, Control, distilled water.

The overall disease incidence in strawberry fruits tended to increase as storage duration progressed, as shown in Figure 3. No disease incidence was observed on the 1st day. By the second day, the control and CO‐treated fruits exhibited 12.5% and 10.61% ± 3.60% disease incidence, respectively, while the CH + CO and AVG + CO treatments showed lower incidences of 6.25% and 8.33% ± 3.60%, respectively. On the 3rd day, the control exceeded its threshold limit at 29.16% ± 3.60%, and CO‐treated fruits reached 25% incidence, with disease symptoms first appearing in fruits treated with CH (8.33% ± 3.60%), AVG (10.41% ± 3.60%), and CH + AVG (6.25%). By Day 4, among coating treatments, CO‐treated fruits showed the highest disease incidence at 35.41% ± 3.60%, whereas CH + AVG treatments recorded the lowest incidence at 18.75%. The incidence of disease may arise from the ongoing enzymatic degradation of the cell wall throughout age, resulting in microfissures that facilitate the invasion of opportunistic fungi. In our investigation, the control exhibited the highest incidence, followed by CO, as CO proved ineffective, which may be due to inadequate adhesion to the hydrophilic fruit surface and partial melting at ambient temperature, resulting in structural fissures that rendered the fruit entirely susceptible to infection. The CH + AVG combination markedly postponed infection, resulting in the lowest overall disease incidence. This is attributed to AVG forming a robust, semi‐permeable barrier that inhibits respiration and obstructs fungal invasion. At the same time, CH functions as a direct antimicrobial agent, utilizing its positive electrical charge to disrupt and eliminate the negatively charged cell membranes of invading fungi.

#### 4.1.5 pH

The impact of various postharvest treatments and the duration of storage on the pH levels of strawberry fruits is detailed in Table 2. Fresh strawberries generally have a pH range of 3.0–3.9, influenced by factors such as cultivar, maturity stage, storage conditions, and microbial activity (Gross et al. 2016). On the first day of storage, the untreated control strawberries had the highest pH of 3.45 ± 0.01, while the CH + AVG treatment had the lowest pH of 3.26 ± 0.01. A gradual increase in pH was observed across all samples during storage. At the conclusion of the storage period, the lowest pH was recorded in the CH + AVG‐treated fruits, followed by the CH, AVG, CH + CO, AVG + CO, and CO treatments, with respective pH values of 3.68 ± 0.02, 3.72 ± 0.01, 3.72 ± 0.02, 3.77 ± 0.02, 3.79 ± 0.01, and 3.81 ± 0.01. No significant differences were observed between the CH and AVG treatments (*p* > 0.05). These findings suggest that postharvest treatments effectively slowed the increase in pH, whereas the control fruits showed a more rapid rise during storage. The treatments based on CO (CO, CH + CO, and AVG + CO) consistently exhibited higher pH values than CH, AVG, and CH + AVG throughout the storage period. Differences in fruit pH resulting from various postharvest treatments are ascribed to the degradation of organic acids during storage, along with microbial and metabolic activities that modify organic acid composition (Temizkan et al. 2019; Mannozzi et al. 2017). During postharvest storage, the pH of strawberries naturally rises due to the continual consumption of organic acids as key substrates for cellular respiration. Edible coatings mitigated this pH shift by creating a semi‐permeable gas barrier that reduces internal oxygen levels, thereby decelerating the fruit's respiration rate and postponing the depletion of organic acids. Sogvar et al. (2016) observed a more pronounced increase in pH in untreated strawberries than in coated strawberries.

**TABLE 2:** Effects of coatings on pH and TA, TSS, and ripening index RI of strawberry fruit during storage periods.

Days of storage	Control	CH	AVG	CO	CH + AVG	CH + CO	AVG + CO
pH							
0	3.2 ± 0.03^p^	3.2 ± 0.03^p^	3.2 ± 0.03^p^	3.2 ± 0.03^p^	3.2 ± 0.03^p^	3.2 ± 0.03^p^	3.2 ± 0.03^p^
1	3.45 ± 0.01^jk^	3.31 ± 0.01^no^	3.34 ± 0.02^mn^	3.4 ± 0.03^klm^	3.26 ± 0.01^op^	3.36 ± 0.03^mn^	3.38 ± 0.01^lmn^
2	3.67 ± 0.01^de^	3.47 ± 0.03^ij^	3.49 ± 0.02^hij^	3.59 ± 0.01^fg^	3.44 ± 0.02^jkl^	3.53 ± 0.02^ghi^	3.55 ± 0.01^gh^
3	3.8 ± 0.02^b^	3.55 ± 0.01^gh^	3.57 ± 0.01^g^	3.7 ± 0.01^de^	3.54 ± 0.01^gh^	3.64 ± 0.01^ef^	3.67 ± 0.01^de^
4	3.88 ± 0.03^a^	3.72 ± 0.01^cd^	3.72 ± 0.02^cd^	3.81 ± 0.01^ab^	3.68 ± 0.02^de^	3.77 ± 0.02^bc^	3.79 ± 0.01^b^
TA							
0	1.08 ± 0.01^a^	1.08 ± 0.01^a^	1.08 ± 0.01^a^	1.08 ± 0.01^a^	1.08 ± 0.01^a^	1.08 ± 0.01^a^	1.08 ± 0.01^a^
1	0.82 ± 0.02^fg^	0.99 ± 0.01^bc^	0.95 ± 0.01^cd^	0.84 ± 0.01^fg^	1.02 ± 0.02^ab^	0.91 ± 0.01^de^	0.87 ± 0.02^ef^
2	0.53 ± 0.02^nop^	0.73 ± 0.02^hi^	0.72 ± 0.04^i^j	0.62 ± 0.01^kl^	0.79 ± 0.02^gh^	0.64 ± 0.01^k^	0.63 ± 0.02^kl^
3	0.36 ± 0.02^r^	0.70 ± 0.02^ij^	0.67 ± 0.02^jk^	0.51 ± 0.01^opq^	0.72 ± 0.02^ij^	0.61 ± 0.01^klm^	0.57 ± 0.01^lmn^
4	0.28 ± 0.01^s^	0.54 ± 0.01^nop^	0.51 ± 0.01^opq^	0.39 ± 0.02^r^	0.55 ± 0.01^mno^	0.49 ± 0.01^pq^	0.46 ± 0.01^q^
TSS							
0	4.21 ± 0.07^r^	4.21 ± 0.07^r^	4.21 ± 0.07^r^	4.21 ± 0.07^r^	4.21 ± 0.07^r^	4.21 ± 0.07^r^	4.21 ± 0.07^r^
1	5.34 ± 0.12^op^	4.88 ± 0.03^q^	4.81 ± 0.08^q^	5.04 ± 0.04^pq^	4.31 ± 0.07^r^	4.92 ± 0.06^q^	4.85 ± 0.10^q^
2	6.19 ± 0.06^ij^	5.71 ± 0.07^mn^	5.52 ± 0.12^no^	6.02 ± 0.10^jkl^	4.92 ± 0.12^q^	5.83 ± 0.10^klm^	5.77 ± 0.05^lmn^
3	7.53 ± 0.10^e^	6.57 ± 0.09^gh^	6.45 ± 0.15^hi^	6.89 ± 0.05^f^	6.14 ± 0.09^jk^	6.85 ± 0.13^fg^	6.75 ± 0.12^fgh^
4	8.38 ± 0.07^a^	7.60 ± 0.14^de^	7.58 ± 0.11^de^	8.2 ± 0.05^ab^	6.9 ± 0.06^f^	7.98 ± 0.13^bc^	7.87 ± 0.10^cd^
RI							
0	3.90 ± 0.08^o^	3.90 ± 0.08^o^	3.90 ± 0.08^o^	3.90 ± 0.08^o^	3.90 ± 0.08^o^	3.90 ± 0.08^o^	3.90 ± 0.08^o^
1	6.48 ± 0.27^klm^	4.90 ± 0.06^mno^	5.02 ± 0.08^mno^	5.95 ± 0.15^lmn^	4.21 ± 0.05^no^	5.37 ± 0.09^mno^	5.55 ± 0.20^mno^
2	11.53 ± 0.57^g^	7.75 ± 0.16^jk^	7.64 ± 0.56^jkl^	9.57 ± 0.31^hi^	6.23 ± 0.31^klm^	9.02 ± 0.21^ij^	9.09 ± 0.24^ij^
3	20.75 ± 1.77^b^	9.32 ± 0.13^ij^	9.60 ± 0.32^hi^	13.46 ± 0.17^ef^	8.46 ± 0.29^ij^	11.18 ± 0.04^gh^	11.65 ± 0.02^g^
4	29.94 ± 1.84^a^	13.99 ± 0.44^ef^	14.65 ± 0.42^de^	20.82 ± 1.17^b^	12.35 ± 0.40^fg^	16.22 ± 0.37^cd^	16.89 ± 0.45^c^

*Note:* Results are expressed as mean ± SD (*n* = 3); means sharing identical letters are not significantly different based on two‐way analysis of variance (ANOVA) followed by Tukey's HSD test (*p* < 0.05).

Abbreviations: AVG + CO, *Aloe vera* gel + coconut oil; AVG, *Aloe vera* gel; CH + AVG, chitosan + *Aloe vera* gel; CH + CO, chitosan + coconut oil; CH, chitosan solution; CO, coconut oil; Control, distilled water.

#### 4.1.6 Titratable Acidity (TA)

The variations in TA of strawberries exposed to various edible coatings are shown in Table 2. Every fruit type has a particular range of acidity that customers appreciate (Khaliq et al. 2019). The high acidity of newly harvested strawberries was evident on the first day, with a TA of 1.08% ± 0.01% across all treatments. The loss of acidity occurred quickly because the higher temperature accelerated microbial and enzymatic breakdown. Significant spoiling was indicated by the control sample's lowest TA (0.28% ± 0.01%) on Day 4. This may occur due to the fruit's natural ripening or because the use of organic acids during respiration accelerates senescence. Díaz‐Mula et al. (2011) assert that the heightened loss of acidity in uncoated fruits results from elevated respiration rates during storage, which modifies the Krebs cycle of organic acids. CH + AVG (0.55% ± 0.01%) showed the highest retention of acidity among the coated treatments, indicating that it successfully inhibited acid‐degrading processes. While CO‐based treatments (CO: 0.39% ± 0.02%, CH + CO: 0.49% ± 0.01%, AVG + CO: 0.46% ± 0.01%) exhibited a relatively faster decline, they were nevertheless superior to the control. CH (0.54% ± 0.01%) and AVG (0.51% ± 0.01%) similarly maintained moderate acidity. As organic acids are substrates for respiratory enzymatic activities, a gradual decrease in TA is anticipated upon harvesting (Saebi et al. 2023). Edible coatings maintain TA by forming a semi‐permeable gas barrier that slows down the fruit's respiration rate, thereby delaying the breakdown of these acids. CO‐based coatings (CO, CH + CO, and AVG + CO) performed similarly to the control samples at the end of the storage period, with CO performance being 0.39% ± 0.02%. Our research indicates that while edible coatings create a semi‐permeable gas barrier that reduces the respiration rate of fruit and consequently delays the degradation of organic acids, CO may not be enough to stop acid deterioration during storage due to its poor adhesion and structural micro‐cracks that failed to restrict gas exchange, despite its antibacterial and antioxidant qualities. AVG coatings probably prevent fruit oxidation and senescence, maintaining higher levels of TA in coated litchi fruit (Ali et al. 2019). Our findings with coated strawberries are consistent with earlier research showing that covering mango fruits with AVG and CH effectively maintains TA content (Seyed et al. 2021).

#### 4.1.7 Total Soluble Solids (TSS)

Table 2 shows that TSS increased gradually in all samples over the course of the experiment and storage time. The increase in TSS can be attributed to water loss, the hydrolysis of cell wall polysaccharides, or the breakdown of starch into simple sugars (Seyed et al. 2021). Conversely, edible coatings can diminish these benefits by restricting respiration and lowering the metabolic rate of the coated fruits (Dong and Wang 2018). Edible coatings may affect respiration and the permeability of O_2_ and CO_2_. As these gases predominantly arise from the breakdown of complex sugars during storage, the use of edible coatings can reduce the degradation process, thereby diminishing the rise in TSS levels (Pinzón et al. 2019). On Day 4, the uncoated (control) fruit showed the highest TSS, 49.76% (8.38 ± 0.07), compared to the initial day. The coated strawberries, on the other hand, achieved a maximum TSS of 48.65% (8.20 ± 0.05) for CO. Because CO coatings were less effective at creating a barrier, water vapor permeability increased, leading to greater water loss and higher TSS levels. The lowest TSS value was observed for the CH + AVG coating, with values of 6.90 ± 0.06 at the end of the storage period. This might be because, at lower concentrations, the AVG coating functioned as a permeable layer while also forming a physical barrier on the fruit's surface (Haider et al. 2022). The findings of Morillon et al. (2002), who observed that AVG had hygroscopic properties, reducing moisture loss and increasing TSS by creating a water barrier between the fruit and its surroundings, are in line with these observations. AVG and CH together produced a formulation that showed synergistic effects, significantly delaying the decrease in TSS, suggesting improved preservation of fruit quality and enhanced composite coating functionality. According to Yin et al. (2019) and Yu et al. (2021), AVG, alone or in combination with CH, helped lower TSS during mango storage, which aligns with our findings for strawberry fruits.

#### 4.1.8 Ripening Index (TSS/TA)

The ripening index progressively increased across all treatments, as expected, as shown in Table 2. On the first day, the slightest decrease in ripening index was 4.21 ± 0.05 for CH + AVG, followed by CH and AVG, while the value was 6.48 ± 0.27, which is 32.02% higher than CH + AVG for uncoated fruits. On Day 4, the uncoated (control) samples demonstrated the greatest ripening indices, 29.94 ± 1.84, which is 86.97% higher than on the initial day. This results from the lack of an edible coating, which elevates the fruit's respiration rate, accelerates ethylene production, and enhances TSS by altering organic acids to starch during ripening (Sophia et al. 2015). During the storage period, following 4 days of treatment, the CH + AVG combination exhibited the minimal rise in ripening index at 12.35 ± 0.40 (68.42%). The protective efficacy of the edible coating may be enhanced by antioxidants in *Aloe vera* leaves, including gallic acid, chlorogenic acid, and catechin, which inhibit rapid free radical release and delay ripening (Álvarez‐Barreto et al. 2023). The second and third‐lowest ripening indices were recorded for CH and AVG, which also contributed to the preservation of strawberry quality. Guillén et al. (2013) reported similar findings for peach and plum fruits treated with AVG, indicating a reduced rate of ripening compared with uncoated fruits. Moreover, edible coatings such as CH have been useful for maintaining strawberry ripening index (Petriccione et al. 2015).

#### 4.1.9 Total Phenolic Content (TPC)

**FIGURE 4:**
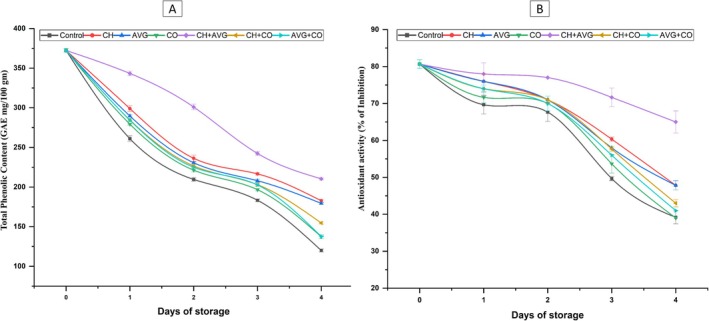
Effects of coatings and storage conditions on total phenolic content of strawberry fruit (A) and antioxidant activity (B) of strawberry fruit during storage period (26°C ± 02°C; 65%–70% RH). AVG + CO, *Aloe vera* gel + coconut oil, AVG, *Aloe vera* gel, CH + VG, chitosan + *Aloe vera* gel, CH + CO, chitosan + coconut oil, CH, chitosan solution, CO, coconut oil, Control, distilled water.

The TPC of strawberries for each coating treatment is shown in Figure 4A. Control samples had the lowest phenolic content by Day 1, whereas CH + AVG had the highest, with values of 343.30 ± 2.88. The phenolic content of non‐coated fruits decreased significantly at the end of the storage period, declining by 67.77%. Fruits coated with CH + AVG, on the other hand, lost significantly less, only 43.59%. When it came to maintaining phenolic content, coatings made with CO were less effective than those made with CH and AVG. The reduction in TPC may result from postharvest cell damage, which facilitates oxygen availability, thereby activating PPO and POD. These enzymes oxidize phenolics into brown polymers, rendering them unquantifiable as “free” phenolics (Tomás‐Barberán and Espín 2001; Qiu et al. 2021). The decrease in TPC may result from the chemical reactivity of phenolics; during storage, they may polymerize, oxidize, or bind to cell wall components, thereby diminishing extractable TPC (Baltacıoğlu et al. 2011; Ghirardello et al. 2016; Jia et al. 2023). Edible coatings protect fruit from moisture and oxygen, hence inhibiting the oxidation of phenolic compounds by enzymes (Aminifard and Mohammadi 2013; Gol et al. 2013). According to Sogvar et al. (2016), phenolic content decreased over time; it was less noticeable in fruits coated with AVG than in those not coated. Similarly, Serrano et al. (2006) found that during postharvest storage, AVG coatings preserved the grapes' phenolic content and other quality characteristics. According to this study (Saleem et al. 2021), CH and ascorbic acid significantly reduced phenolic loss, which may be especially advantageous for strawberries stored in cold storage. Our results showed that phenolic content was higher in fruits coated with CH + AVG than in fruits that were either untreated or coated with AVG. Begum et al. (2023) found that combining CH with AVG successfully prevented a decrease in phenolic content in fruits. Furthermore, Seyed et al. (2021) discovered that CH coatings and AVG had a favorable effect on the preservation of phenolic content in mangoes over time.

#### 4.1.10 Total Antioxidant Capacity

Figure 4B shows each sample's antioxidant capacity. Over time, both coated and control fruits' DPPH scavenging activity progressively declined. The control fruits showed a faster rate of decline, whereas fruits coated with CH + AVG exhibited the highest DPPH activity at the end of the storage period, with 54.30% ± 0.51%. By contrast, the DPPH activity of the uncoated fruits was considerably lower, measuring 30.40% ± 0.34%. Throughout the experiment, this pattern held: the non‐coated fruits exhibited the lowest antioxidant activity, while the CH + AVG, AVG, and CH coatings exhibited the highest. Senescence and degradation are likely responsible for the drop in antioxidant activity observed during storage. This reduction may be the consequence of antioxidants' diminished ability to preserve fruit quality, delay deterioration, and inhibit the enzymes that break them down. The total antioxidant activity in strawberries is significantly reliant on the availability of potent oxygen radical scavengers, including vitamin C and phenolic compounds (Tulipani et al. 2008). During postharvest storage, fruit undergoes environmental stress, including water loss, leading to rapid accumulation of reactive oxygen species (ROS), such as superoxide and H_2_O_2_. To protect cellular tissues from oxidative damage, the fruit continuously depletes its non‐enzymatic antioxidant reserves. This continuous use depletes the remaining scavengers, leading to a documented reduction in measurable antioxidant activity over time (Meitha et al. 2020; Liu et al. 2019; Xu et al. 2021). AVG coatings strengthen the antioxidant system, which may increase the fruit's resilience to degradation, according to Hu et al. (2005). Sogvar et al. (2016) found similar results, noting that fruits coated with ascorbic acid and AVG exhibited a less pronounced decline in antioxidant activity. Additionally, Hu et al. (2005) showed that AVG strengthens tissues' resistance to deterioration by improving their antioxidant system and capacity to scavenge free radicals. Shah and Hashmi (2020) and Seyed et al. (2021) found that mangoes treated with CH and AVG maintained DPPH scavenging activity, which aligns with our findings.

#### 4.1.11 Total Yeast and Mold Count (TYMC)

**FIGURE 5:**
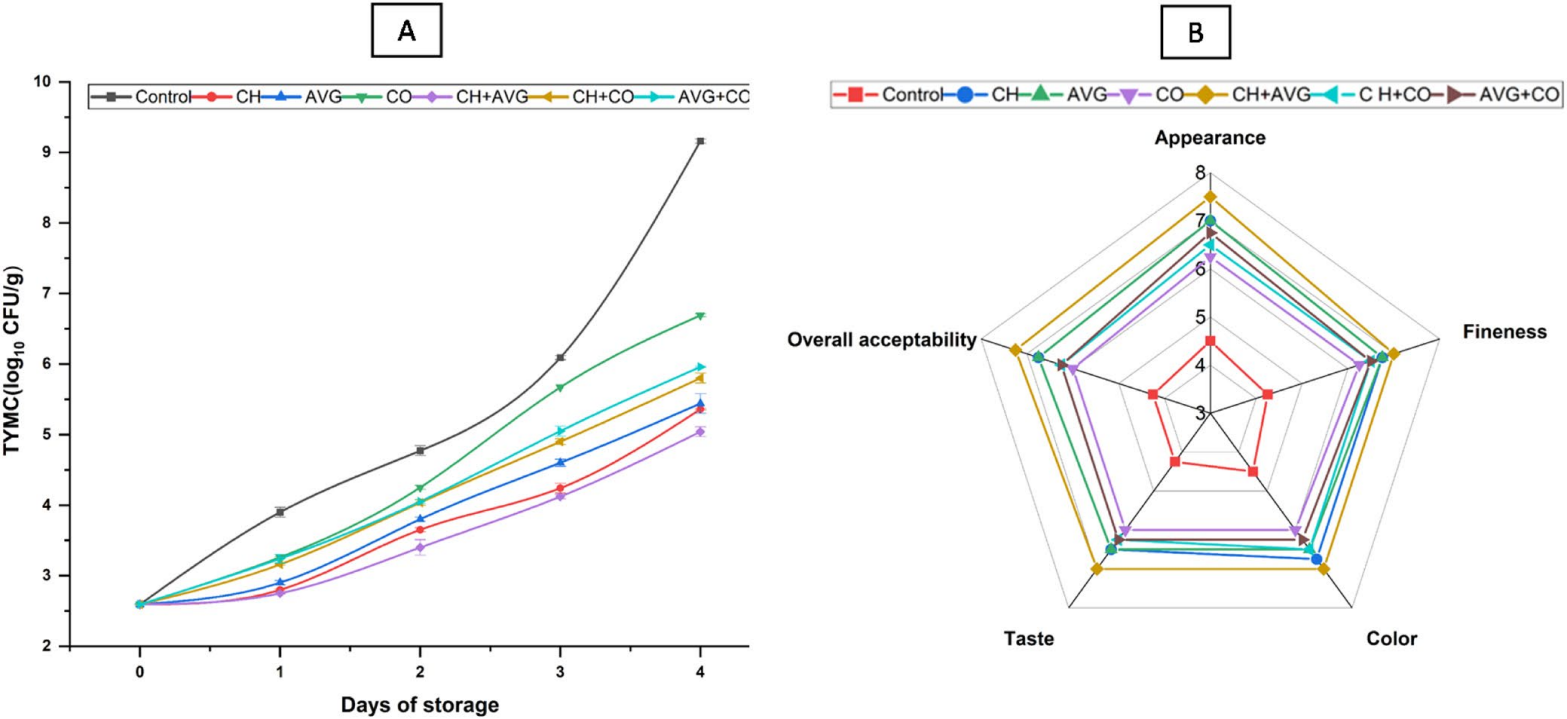
Effects of coatings on total yeast and mold count of strawberry fruit (A) and sensory assessment scores (B) at 26°C ± 02°C (65%–70% RH) during storage period. AVG + CO, *Aloe vera* gel + coconut oil, AVG, *Aloe vera* gel, CH + VG, chitosan + *Aloe vera* gel, CH + CO, chitosan + coconut oil, CH, chitosan solution, CO, coconut oil, Control, distilled water.

The TYMC for strawberries subjected to various treatments is illustrated in Figure 5A. Strawberries are especially susceptible to microbial spoilage caused by *Botrytis cinerea* due to their elevated water content and physiological activities (Khodaei et al. 2021). On the final day of storage, strawberries treated with CH + AVG exhibited the lowest TYMC (5.04 ± 0.07), while the control samples recorded the highest TYMC (9.16 ± 0.03). The TYMC values for CH and AVG treatments were 5.36 ± 0.01 and 5.44 ± 0.14 log_10_ CFU/g, respectively, with no statistically significant difference (*p* > 0.05), indicating comparable antimicrobial effectiveness between the two treatments at this point. As noted by Omorodion and Chinwo (2022), fruits with elevated water activity, higher sugar levels, and pH below 4.4 promote the proliferation of osmophilic yeasts and xerotolerant fungi during storage. Common spoilage microorganisms found in strawberries include total aerobic mesophilic bacteria, molds, and yeasts; acceptable levels are below 10^6^ CFU/g (Popescu et al. 2022; Perez‐Vazquez et al. 2023). CH coatings and chitosan‐based edible films have been reported to inhibit the growth of major fungal pathogens, such as *Cladosporium* sp. and *Rhizopus* sp., while also exhibiting antibacterial activity that extends shelf life in strawberries (Rodriguez et al. 2003; Park et al. 2005; Velickova et al. 2013). Likewise, Perdones et al. (2012) found that the combination of CH and lemon essential oil effectively reduced fungal activity in strawberries stored at 5°C. After a 12‐day storage period, Vu et al. (2011) observed that limonene and CH exhibited a more potent antifungal effect compared to the control sample. AVG has been reported to exhibit stronger antibacterial activity against Gram‐positive bacteria than against Gram‐negative bacteria, and it is also effective in inhibiting fungal pathogens such as *Penicillium digitatum*, *Botrytis cinerea*, and *Rhizopus stolonifer* (Saritha et al. 2010; Navarro et al. 2011). The antibacterial properties are believed to stem from AVG constituents, including saponins, acemannan, and anthraquinone derivatives, which reduce the growth of microorganisms (Martínez‐Romero et al. 2006). Our results are consistent with previous studies on sweet cherries and table grapes treated with AVG, which reported reductions in populations of yeast, mold, and mesophilic aerobic bacteria during storage (Martínez‐Romero et al. 2006; Valverde et al. 2005). We observed that AVG and CH collaborated effectively to inhibit microbial growth in strawberries during storage.

### 4.2 Sensory Evaluation

The sensory evaluation results for strawberries treated with different coatings are shown in Figure 5B. Consumer acceptance or rejection of a food product is largely influenced by sensory characterization (Moskowitz et al. 2012). According to the findings, strawberries coated with CH + AVG consistently performed better than all other treatments across all sensory categories. The greatest ratings on the 9‐point hedonic scale were given to CH + AVG, demonstrating its exceptional capacity to maintain the sensory quality of strawberries while they are being stored. The CH and AVG coatings, on their own, also performed well, scoring higher than the other treatments, especially in fineness and appearance. By creating semi‐permeable barriers that inhibited oxidation and moisture loss, these coatings probably contributed to the fruit's continued firmness and freshness. CO‐based coatings (CO, CH + CO, and AVG + CO), on the other hand, scored comparatively lower on every sensory metric. Specifically, the CO‐only treatment resulted in decreased taste and overall acceptability, which may have been due to an uneven coating caused by the oil's hydrophobic structure or its low antioxidant capacity. Even so, the coconut‐based coatings performed better than the control, which consistently scored lowest throughout storage due to obvious spoilage and textural deterioration. All effects considered, the CH + AVG combination worked best to preserve the strawberries' sensory qualities over time.

### 4.3 Analyzing the Multivariate Statistical Relationships

#### 4.3.1 Principal Component Analysis (PCA)

**FIGURE 6:**
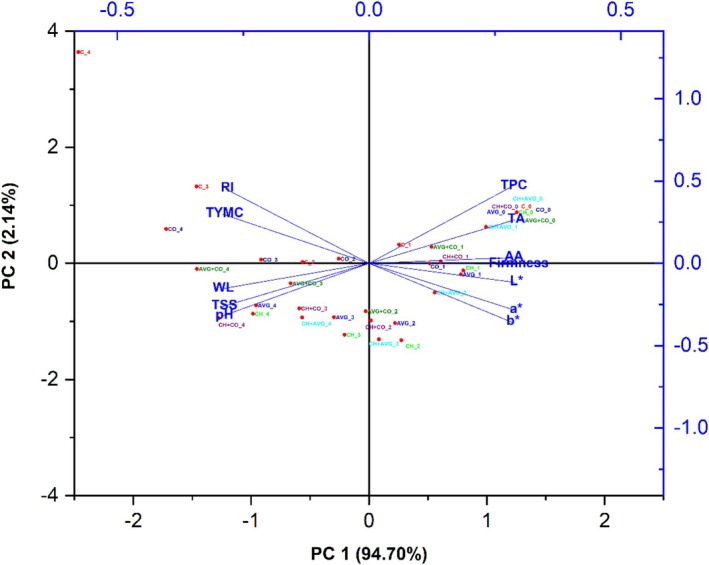
Principal component analysis of physiological quality attributes, bioactive compounds, antioxidant activity, and microbial analysis of different treated and untreated strawberry fruit.

PCA was performed to visualize the interrelationship between the quality parameters and treatments of coated strawberries during storage. The first two principal components, PC1 and PC2, explained 94.70% and 2.14% of the total variance, respectively, in Figure 6. Antioxidant activity, firmness, color properties, TA, TPC, and firmness all had positive correlations with PC1, indicating that samples with higher PC1 scores maintained superior physicochemical quality throughout time. Better quality preservation was indicated by coating treatments that clustered on this positive axis during earlier storage days (Days 0, 1, 2), including CH + AVG, CH, AVG, CH + CO, and AVG + CO. A negative correlation between PC1 and variables such as weight loss, pH, TSS, RI, and TYMC displayed significant loading on the negative axis. This suggests that the fruit's quality has declined. The proximity of the firmness, antioxidant activity, TPC, and TA vectors on the positive side of PC1 suggests a high positive association between these quality metrics. Regardless of biochemical or textural characteristics, the color parameters appear to be densely clustered, forming a distinct cluster that demonstrates their strong interdependence in maintaining visual quality. TSS, pH, and weight loss percentage all cluster together on the negative side of PC1, highlighting their association with physicochemical degradation. Furthermore, the upper‐left quadrant shows distinct groups for the ripening index and TYMC, indicating their involvement in the ripening index and microbial spoiling. It is interesting to note that CO treatment on Days 0 and 1 seemed closer to quality‐retaining characteristics, but by Days 2, 3, and 4, it decreased quickly and began to cluster with spoilage signs. This confirms that CO by itself is not very effective for long‐term preservation. Overall, the PCA demonstrates that composite coatings, particularly CH + AVG, are more effective at maintaining the postharvest quality of strawberries.

#### 4.3.2 Correlation Analysis

**FIGURE 7:**
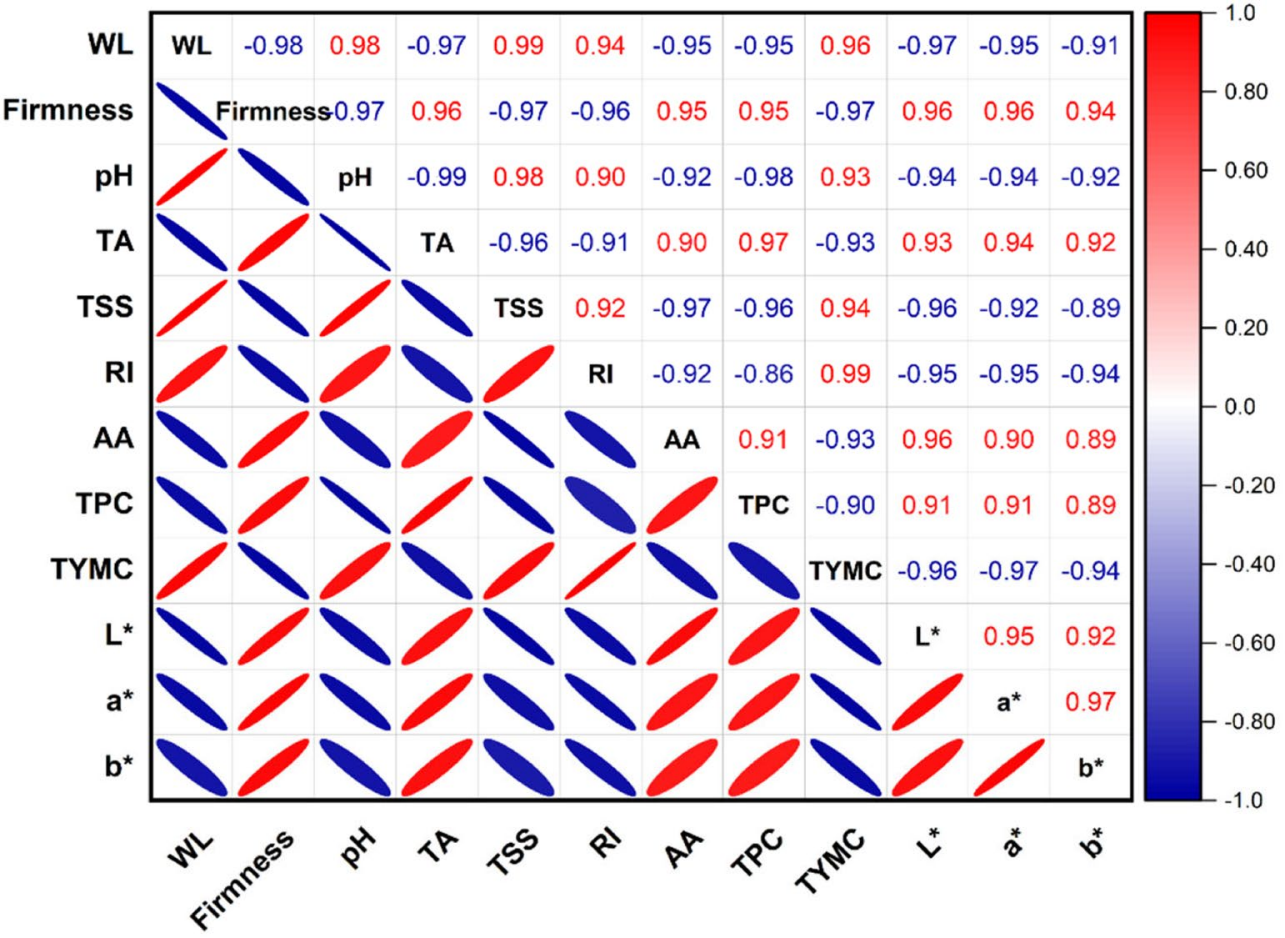
Pearson's correlation analysis among physicochemical attributes, bioactive, antioxidant activity, microbial load, and color parameters of coated and uncoated strawberry fruit during storage; AA, antioxidant activity; RI, ripening index; TA, titratable acidity; TPC, total phenolic content; TSS, total soluble solids; TYMC, total yeast and mold count; WL, weight loss.

The Pearson correlation method was used to assess the relationship between strawberry quality parameters during storage, as outlined by Yang et al. (2020), as shown in Figure 7. Firmness and weight loss had a highly significant negative association (*r* ^2^ = −0.98), but weight loss and TYMC had a substantial positive correlation (*r* ^2^ = 0.96). Chen et al. (2016) claim that fruit's cell membrane structure is weakened by water stress, which also causes water loss, resulting in a reduction in firmness. According to Lakey and Anderluh (2016), cell membrane structure damage is a major contributing element to microbial attack. Additionally, the color parameters showed a substantial positive correlation with weight loss, with *r* ^2^ values of −0.97 for L*, −0.95 for a*, and −0.91 for b*. These results are in line with those of Palma‐Orozco et al. (2012), who proposed that faster weight loss accelerates flesh browning, leading to a decrease in fruit color. Weight loss was also strongly positively correlated with TSS (*r* ^2^ = −0.99), suggesting that strawberries with higher TSS content are more likely to cause weight loss. Since pH and acidity are inversely correlated, it is not surprising that pH and TA showed an almost perfect negative correlation (*r* ^2^ = −0.99).

#### 4.3.3 Hierarchical Clustering Analysis (HCA)

**FIGURE 8:**
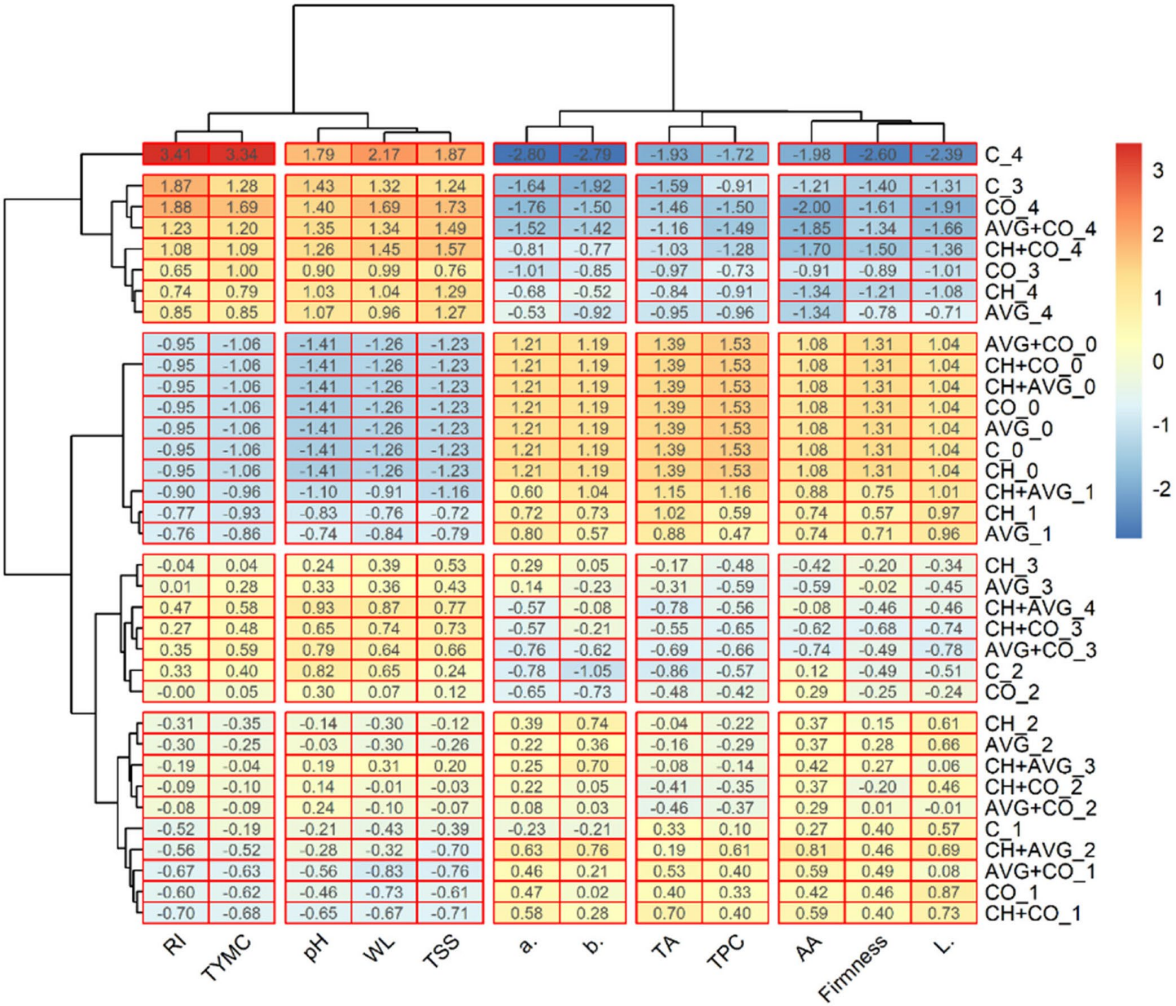
Hierarchical cluster analysis of physicochemical attributes, bioactive, antioxidant activity, microbial load, and color parameters of coated and uncoated strawberry fruit during different storage periods.

The treatments and 12 quality parameters were grouped using hierarchical cluster analysis; the ideal number of clusters was found to be five, as illustrated in Figure 8. Based on their daily groupings, the treatments were categorized into five clusters (I, II, III, IV, and V). Cluster I consists of 10 treatments: CH_2, AVG_2, CH + AVG_3, CH + CO_2, AVG + CO_2, C_1, CH + AVG_2, AVG + CO_1, CO_1, and CH + CO_1. Cluster II: CH_3, AVG_3, CH + AVG_4, CH + CO_3, AVG + CO_3, C_2, and CO_2. Cluster III: CH_1, AVG_1, CH + AVG_1, CH_0, C_0, AVG_0, CO_0, CH + CO_0, AVG + CO_0, and CH + AVG_0. Cluster IV: C_3, CO_4, AVG + CO_4, CH + CO_4, CO_3, CH_4, and AVG_4. Finally, Cluster V consists of a single treatment: C_4. The coating treatments of strawberry fruits belonging to cluster II exhibited significantly lower TA, color values, antioxidant activity, and firmness compared to treated samples in clusters I and III, with statistical significance at *p* < 0.05. These findings indicate that strawberries grouped in clusters I and III demonstrated superior acid retention, antioxidant stability, color retention, and firmness during storage, particularly at the beginning of the storage period. The results suggest that treatments in this cluster (I and III) were more effective in delaying metabolic degradation and preserving bioactive compounds over time. Quality indicators such as antioxidant activity, firmness, and L* form cluster I; TA and TPC form cluster II; and a* and b* form cluster III. Finally, the remaining indicators, particularly the fruit pH, weight loss, and TSS from cluster IV and RI and TYMC, were grouped in cluster V, aligning with PCA results.

### 4.4 Advanced Analytical Approaches for Prediction and Treatment Ranking

#### 4.4.1 Random Forest Classification for Predicting Important Variables Involves of Coating Differentiation

**FIGURE 9:**
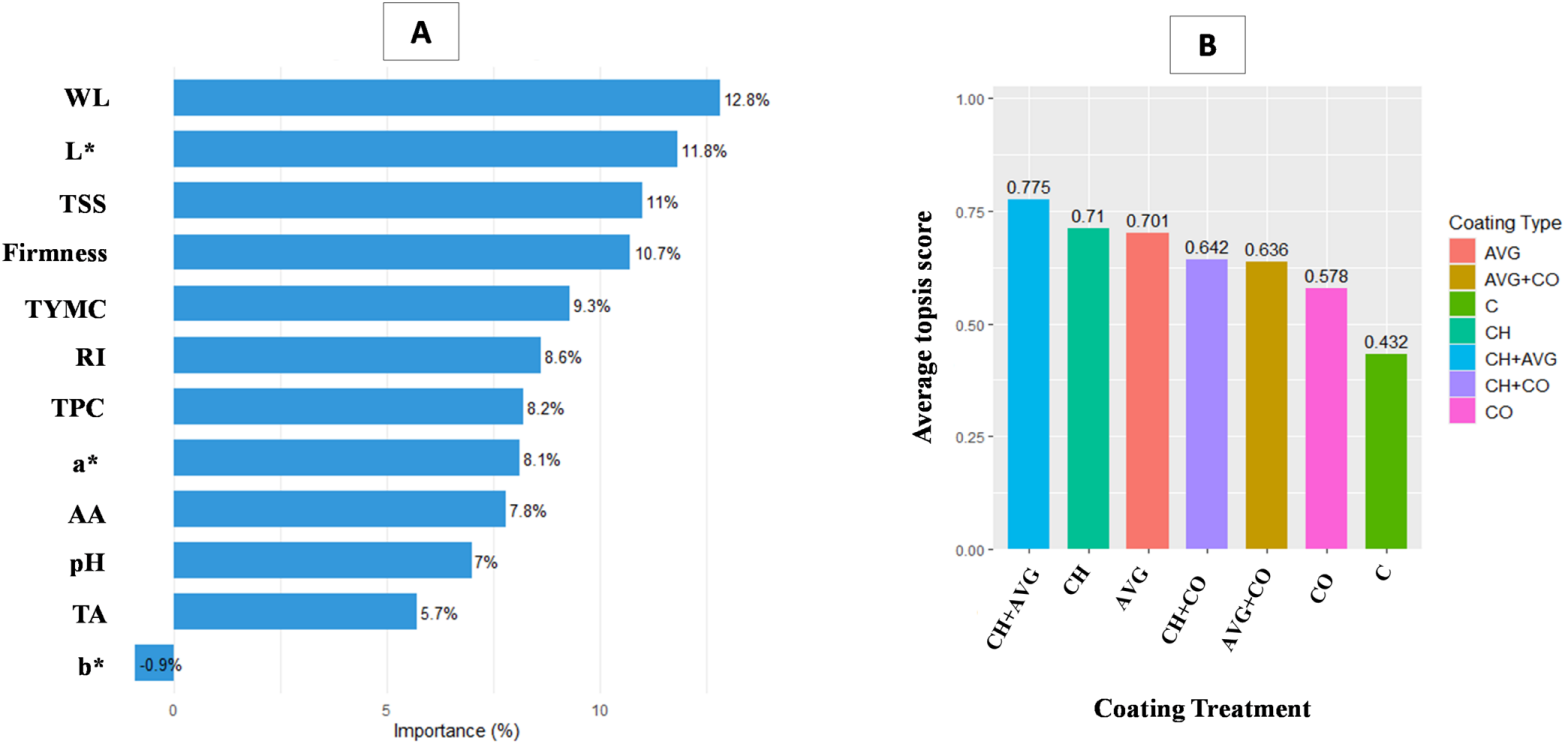
Random forest classification (A) and TOPSIS analysis (B) of treated strawberry fruit.

Random Forest classification was performed to ascertain the most significant quality characteristics for differentiating coating treatments. The Random Forest classification results shown in Figure 9A indicate that water loss, which contributed 12.8% to the model's predictive performance, was the most significant variable among the measured parameters. L* at 11.8%, TSS at 11%, and hardness at 10.7% were closely followed, suggesting that the samples' optical and physical characteristics were crucial for identifying coatings. Significant influence was also demonstrated by TYMC (9.3%) and RI (8.6%), indicating that ripening index and microbiological stability are pertinent categorization variables. While pH and TA were somewhat less important (7% and 5.7%, respectively), other characteristics, such as a* and antioxidant activity, contributed substantially, ranging from 8.2% to 7.8%. It is interesting to note that the variable b* contributed negatively (−0.9%), suggesting that it had little to no bearing on the coating classification. These results reveal that coating efficiency is closely related to the Random Forest model, which mainly depends on characteristics of product appearance, physicochemical quality, and textural integrity.

#### 4.4.2 TOPSIS Analysis for Multi‐Criteria Ranking of Coating Treatments

The best coating treatment for increasing strawberry shelf life was identified using technique for order of preference by similarity to ideal solution (TOPSIS) approach in Figure 9B. The following characteristics were identified as positive (higher values are preferred): firmness, TA, color parameters (L*, a*, b*), antioxidant activity, and TPC. However, the following factors were considered negative: pH, TSS, ripening index, TYMC, and weight loss. Among the studied coatings, the CH + AVG coating proved to be the most effective treatment for strawberries, with a TOPSIS score of 0.775, successfully managing weight loss, TYMC, and TSS while maintaining vital nutrients, including antioxidants, and sensory qualities during storage. According to these findings, the shelf life of fresh strawberries was significantly extended by coating treatments, with CH + AVG as the best coating.

## 5 Conclusion

The study assessed the efficacy of various edible coatings in maintaining the postharvest quality of strawberries (Fragaria *× ananassa*) stored under ambient conditions. This study shows that combining CH and AVG is an effective postharvest technique for improving strawberry storage quality for up to 4 days. Fruit firmness was maintained, weight loss decreased significantly, and important physicochemical characteristics, such as pH, TA, and TSS, were maintained, all of which were attributed to the CH + AVG coating. In addition, it preserved the skin's appearance and limited the growth of microorganisms, while preventing the deterioration of vital bioactive compounds, such as total phenolics and antioxidant capacity. AVG and CH together provided greater preservation benefits than either coating alone, especially when stored at ambient conditions. These edible coatings present a viable, eco‐friendly way to extend the shelf life of fruit in light of the growing emphasis on food safety and quality. However, the study was limited to ambient storage conditions and did not evaluate refrigerated storage; therefore, future research should investigate the performance of coating under low‐temperature conditions for potential cold‐chain and long‐distance transport applications. In addition, the characterization of the film's mechanical properties, barrier properties, and molecular arrangement was beyond the scope of the present work and remains a central focus of our future research.

## Author Contributions

**Gazi Mohammad Tasnimul Karim:** conceptualization, methodology, software, data curation, formal analysis, visualization, writing – original draft, writing – review and editing. **Mrityunjoy Biswas:** conceptualization, writing – review and editing, investigation, validation, supervision, resources. **Soheli Sultana:** data curation, writing – review and editing. **Munmun Nahar:** conceptualization, methodology, software, data curation, visualization, writing – original draft, writing – review and editing, formal analysis.

## Funding

The authors have nothing to report.

## Ethics Statement

The sensory evaluation conducted in this study involved strawberry fruit treated with edible coatings. No human or animal subjects were exposed to any invasive procedures or risks.

## Conflicts of Interest

The authors declare no conflicts of interest.

## Data Availability

Data will be made available on request.
